# Rapid identification of the predominant azole-resistant genotype in *Candida tropicalis*

**DOI:** 10.1093/femsyr/foae025

**Published:** 2024-10-21

**Authors:** Kuo-Yun Tseng, Yu-Chieh Liao, Yin-Zhi Chen, Feng-Chi Chen, Feng-Jui Chen, Huey-Kang Sytwu, Li-Yun Hsieh, Chung-Yu Lan, Hsiu-Jung Lo

**Affiliations:** Taiwan Mycology Reference Center, Institute of Infectious Diseases and Vaccinology, National Health Research Institutes, Miaoli 350401, Taiwan, ROC; Institute of Molecular and Cellular Biology, National Tsing Hua University, Hsinchu 300044, Taiwan, ROC; Institute of Population Health Sciences, National Health Research Institutes, Miaoli 350401, Taiwan, ROC; Taiwan Mycology Reference Center, Institute of Infectious Diseases and Vaccinology, National Health Research Institutes, Miaoli 350401, Taiwan, ROC; Institute of Population Health Sciences, National Health Research Institutes, Miaoli 350401, Taiwan, ROC; Taiwan Mycology Reference Center, Institute of Infectious Diseases and Vaccinology, National Health Research Institutes, Miaoli 350401, Taiwan, ROC; Taiwan Mycology Reference Center, Institute of Infectious Diseases and Vaccinology, National Health Research Institutes, Miaoli 350401, Taiwan, ROC; Taiwan Mycology Reference Center, Institute of Infectious Diseases and Vaccinology, National Health Research Institutes, Miaoli 350401, Taiwan, ROC; Institute of Molecular and Cellular Biology, National Tsing Hua University, Hsinchu 300044, Taiwan, ROC; Department of Life Science, National Tsing Hua University, Hsinchu 300044, Taiwan, ROC; School of Medicine, National Tsing Hua University, Hsinchu 300044, Taiwan, ROC; Taiwan Mycology Reference Center, Institute of Infectious Diseases and Vaccinology, National Health Research Institutes, Miaoli 350401, Taiwan, ROC; School of Dentistry, China Medical University, Taichung 404328, Taiwan, ROC; Department of Biological Science and Technology, National Yang Ming Chiao Tung University, Hsinchu 300093, Taiwan, ROC

**Keywords:** *Candida tropicalis*, MLST, xylose reductase, *XYR1*, *SNQ2*

## Abstract

*Candida tropicalis* is a leading cause of nonalbicans candidemia in tropical/subtropical areas and a predominant genotype of azole-resistant *C. tropicalis* clinical isolates belongs to clade 4. The aim of this study was to reveal markers for rapidly identifying the predominant azole-resistant *C. tropicalis* genotype. We analysed *XYR1*, one of the six genes used in the multilocus sequence typing analysis, and *SNQ2*, an ATP-binding cassette transporter in 281 *C. tropicalis*, including 120 and 161 from Taiwan and global areas, respectively. Intriguingly, the first 4-mer of codon sequences ATRA of CTRG_05978 (96/119 versus 21/162, *P* < .001, at phi = 0. 679) and the *SNQ2* A2977G resulting in amino acid I993V alternation (105/118 versus 12/163, *P* < .001, at phi = 0.81) was significantly associated with the clade 4 genotype. The sensitivity and specificity of the clade 4 genotype detection with a combination of SNPs of CTRG_05978 and *SNQ2* were 0.812 and 0.994, respectively, at phi = 0.838. Furthermore, we successfully established a TaqMan SNP genotyping assay to identify the clade 4 genotype. Our findings suggest that to improve the management of *C. tropicalis* infections, rapidly identifying azole-resistant *C. tropicalis* by detecting SNPs of CTRG_05978 and *SNQ2* is promising.

## Teaser

New methanogens from arthropod guts demonstrate significant genome reduction but also presence of high-affinity terminal oxidases.

## Introduction

*Candida* spp. are typically harmless commensal yeasts found in various environmental, human, and other mammalian sources (Yang et al. 2012, Mareković et al. 2021, Zheng et al. 2021). Within humans, they play a vital role as a part of the microflora present on the skin, oral, and vaginal mucosa, as well as within the gastrointestinal tract. However, in case of severely compromised immunity, they can transform into opportunistic pathogens, leading to illness and even lethality (Pfaller and Diekema 2010). *Candida* infections have increased significantly and have been associated with 0.75 million deaths annually, posing serious public health problems (Thune et al. 2016). Among *Candida* spp., *Candida albicans* is the most frequently isolated in hospital settings (Yang et al. 2010, Mareković et al. 2021). However, the trend has changed toward the more treatment-resistant nonalbicans *Candida* spp. (Yang et al. 2004, Pfaller et al. 2019, Kumar et al. 2022). The prevalence of these species may vary based on the geographical location. For example, *Candida tropicalis* stands out among the nonalbicans *Candida* species as the second or even the most prevalent pathogenic yeast in some regions, such as Asia and Latin America (Colombo et al. 2006, Zhou et al. 2016).

Molecular epidemiological investigation of *C. tropicalis* can be instrumental in identifying its epidemic genotype, potential modes of transmission, genetic composition of drug-resistant isolates, biological habitats, and population structure. The multilocus sequence typing (MLST) has been developed to characterize population structures within a species and distinguish geographical origins (Tavanti et al. 2005, Odds and Jacobsen 2008). The combinations of alleles at different loci yield unique diploid sequence types (DSTs), which enable differentiation and classification of genetic relatedness among isolates. The current MLST approach for *C. tropicalis* utilizes six genes, including *ICL1, MDR1, SAPT2, SAPT4, XYR1*, and *ZWF1a* (http://pubmlst.org/ctropicalis/). Moreover, MLST has been extensively employed to assess the clonality of *C. tropicalis* and ascertain various traits, such as fluconazole susceptibility, hospital-based origins, and anatomical sources. Finally, the allelic profiles of isolates can be compared by utilizing the online MLST database to explore diverse geographical origins, drug resistance transmission, and genetic variation patterns (Yang et al. 2012, Dougue et al. 2022, Zhou et al. 2022).

Of interest, MLST has been also exerted to uncover that fluconazole-resistant *C. tropicalis* isolates share the same clade 4 genotype (Tseng et al. 2022, Zhou et al. 2022). These findings underscore the utility of MLST typing as a method for distinguishing drug-resistant *C. tropicalis* isolates. Although MLST offers an easy application in determining genotype of *C. tropicalis*, a need for rapid identification and more comprehensive characterization of drug-resistant isolates remains vital in the realms of diagnosis and antifungal stewardship. Therefore, this current study endeavored to elucidate the genotypes of one of *XYR1* genes, CTRG_05978, and *SNQ2*, which is situated 25 kb upstream from CTRG_05978 and has been linked to drug susceptibility in *Nakaseomyces glabratus* (*Candida glabrata*) and *Candida auris* (Torelli et al. 2008, Wasi et al. 2019) were the potential markers that enable the rapid identification of azole-resistant *C. tropicalis*, contributing to the prevention of further dissemination of the predominant clade 4 isolates.

## Materials and methods

### Strains isolation

*Candida tropicalis* collected from Taiwan surveillance of Antimicrobial Resistance of Yeasts (TSARY) studies were described previously (Yang et al. 2012, Zhou et al. 2016). All yeast strains were identified by the sequences of the rRNA as described (Leaw et al. 2007). To make sure that we have representative population of *C. tropicalis* from our collection to begin with, we analysed at least 10 strains (if there were) each of 11 most common clades including 37 strains having whole-genome data. A total of 120 *C. tropicalis* strains, including ATCC750 and 119 from TSARY (Table S3) were analysed initially to identify potential markers. To verify our findings, we further analysed 161 strains including all clade 4 whole-genome data that we could find from the NCBI database along with 10 other clades that contain the most DST members.

### DNA extraction and MLST analysis of *C. tropicalis* isolates

Genomic DNA of each isolate was extracted using a Yeast Genomic DNA Kit (Geneaid) according to the manufacturer’s instructions, and DNA was quantified using the Qubit fluorometer (ThermoFisher Scientific). The DNA fragments of six genes—*ICL1, MDR1, SAPT2, SAPT4, XYR1*, and *ZWF1a*—were amplified and sequenced as described in previous studies (Yang et al. 2012, Zhou et al. 2022). The resultant sequences were aligned with BioNumerics 3.0 (Applied Maths, Kortrijk, Belgium) and compared with those in the database of *C. tropicalis* (http://pubmlst.org/website) to determine the level of sequence identities (DST). The primers used are listed in Table S1.

### Whole genome sequencing

The high molecular weight DNAs were used to construct a multiplexing nanopore sequencing library using the Oxford Nanopore Technologies (ONT) Ligation Sequencing Kit (SQK-LSK109) and Native Barcoding Expansion Kit (EXP-NBD104), following ONT’s instructions. A standard 72-hour sequencing script was executed on MinKNOW; raw reads were collected then base called and demultiplexed using the standalone application, Guppy.

### Investigation of *XYR1* by nanopore

The raw reads of 17 *C. tropicalis* isolates were aligned to the *XYR1* gene (XM_002546469.1) using minimap2 (Li 2018) to produce reads containing *XYR1*. The *XYR1*-containing reads were then de novo assembled using SMARTdenovo (version 1.0) (Liu et al. 2021) to form contigs.

The assembled *XYR1*-containing contigs were further polished with Medaka (version 1.5.0) using the raw reads and rearranged to maintain the same orientation as the *C. tropicalis* MYA-3404 genomic scaffold supercont3.11 (NW_003020040.1). The sequence analyses for the 17 *XYR1*-containing contigs revealed a similar schematic representation of the two *XYR1* genes compared to the MYA-3404 strain.

### Multiple sequence alignments and evolutionary distances

After designing the primer sequences for sequencing the partial sequences of CTRG_05978 and CTRG_05993, we analysed 120 strains of *C. tropicalis*, including ATCC750, and 119 from clinical sources. The *XYR1* DNA sequence was aligned using ClustaIW multiple alignment in MEGA 10 (Kumar et al. 2018). Pairwise genetic distances were separated into intraspecific and interspecific parameters and calculated to characterize both intra- and interspecific variation within and between CTRG_05978 CDS and CTRG_05993 CDS by using MEGA10. The statistical significance (*P*-value), Wilcoxon rank sum, and signed-rank tests were determined by R software version 4.1.2 (Wilcox.test package).

### TaqMan SNP genotyping

The TaqMan SNP genotyping assay was performed using TaqMan Genotyping Master Mix (Applied Biosystems) and the ABI QuantStudio^™^ 6 Flex System (Applied Biosystems), following the manufacturer’s instructions. Thermal cycle conditions in CTRG_05978 were as follows: 1 cycle of 95°C for 10 min, 40 cycles of 95°C for 15 s, 54°C for 20 s and 60°C for 1 min, and 1 cycle of 60°C for 1 min; and in *SNQ2* were as follows: 1 cycle of 95°C for 10 min, 40 cycles of 95°C for 15 s, 60°C for 1 min, and 1 cycle of 60°C for 1 min.

### Statistical tests

SPSS software for Windows (version 12.0) was used to analyse the data. The null hypothesis that the frequencies of the MLST genome type and CTRG_05978 start codon region type are equal was investigated by the chi-square (χ^2^) test.

The chi-squared or Fisher’s exact test with 1-tailed correction was applied for categorical variables. Logistic regression was applied to assess the independent effects of factors with values less than 0.05 in univariate analysis. A *P*-value less than .05 was considered significant.

## Results

### Two *XYR1* genes were likely to be ancestral duplications

**Figure 1.**
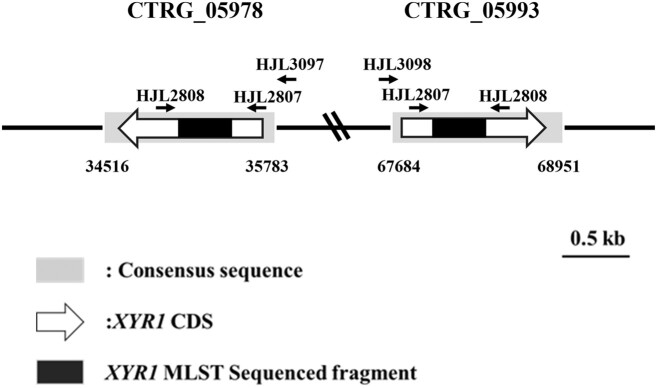
Schematic representation of the two *XYR1* genes. The direction of the arrows indicates the direction of two *XYR1* genes. Since they are high consensus, in order to differentiate the sequence of MLST fragments of two *XYR1* genes, we employed primer set HJL2808/HJL3097 and HJL2808/HJL3098 for CTRG_05978 and CTRG_05993, respectively.

**Figure 2.**
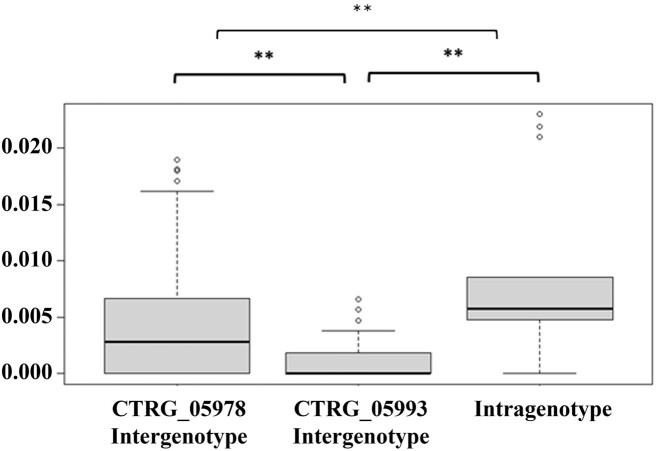
The diversity of two *XYR1* genes. The distance of intragenotype and intergenotype for two *XYR1 genes*. In this box plot of genetic distances, including intra and intergenotype distance for CTRG_05978 and CTRG_05993, the box represents the interquartile range, with the lines extending the range of the data. Points outside the range are mild outliers, with values greater than 1.5 × the upper bound of the interquartile range. **P-value < .01.

Two *XYR1* genes, CTRG_05978 and CTRG_05993 (GenBank accession number: NW_003020040.1), on chromosome VI with ~30 kb apart from each other in opposite orientations were detected (Fig. 1). A distance matrix analysis, including intra and intergenotype distances for CTRG_05978 and CTRG_05993, was performed using MEGA X software (Kumar et al. 2018). The boxplots of the distance index values for the CTRG_05978 intergenotype cohorts (median = 0.003, mean ± SD = 0.004 ± 0.005), the CTRG_05993 intergenotype cohorts (median = 0, mean ± SD = 0.002 ± 0.001), and the intragenotype cohorts (median = 0.005, mean ± SD = 0.009 ± 0.007) were generated. Notably, the largest distance was obtained from the intragenotype comparisons (0–0.022), while the distances within the CTRG_05993 intergenotype cohorts showed relatively low values (0–0.005) (Fig. 2 and Fig. S1). The fact that the intragenotype distance was greater than the individual intergenotype distances of CTRG_05978 and CTRG_05993 suggested that these two alleles may be ancestral duplications.

### The SNP of the translation initiation codon of CTRG_05978 associated with the predominant clade 4 azole-resistant *C. tropicalis* genotype

Due to the higher diversity of CTRG_05978, we further analysed its sequence. We found several SNPs in the translation initiation codon of CTRG_05978 (Tables S2 and S3). One of these SNPs with G to A substitution in the start codon resulted in a loss-of-function mutation. We then conducted a more comprehensive analysis of the first 60 nucleotides in the codon sequences of CTRG_05978 across all 120 isolates. Notably, ~82.5% (99 out of 120) of the isolates possessed at least one allele of CTRG_05978 that harbored a loss-of-function mutation in Xyr1.

Furthermore, the first 4-mer of CTRG_05978 open reading frame could be classified into seven different sequence types (Table S2). Interestingly, an association between the sequence of CTRG_05978 and DST by MLST was observed (Table 1 and Table S3). The majority of clade 4 strains tested, with the exception of YM180542 (DST506) and YM180921 (DST508), exhibited the genotype IV (ATRA) sequence (36/48 versus 2/72, *P* < .001). This significant association between the sequence of CTRG_05978 and the clade 4 genotype was further confirmed by logistic regression analysis (*P* < .001; OR = 96.92) (Table S4).

**Table 1.** The association of genotype and sequence types of CTRG_05978 among 120 *C. tropicalis* isolates.

	Sequence type of CTRG_05978							
Clade	I (ATAA)	II (ATGA)	III (ATGT)	IV (ATRA)	V (ATGW)	VI (ATRW)	VII (ATWA)	Total
1	4	0	0	0	0	0	0	4
2	0	0	12	0	0	0	0	12
3	5	4	0	3	0	2	1	15
4	2	0	0	36	0	0	0	38
5	18	0	0	0	0	1	0	19
6	1	0	1	2	1	7	0	12
7	2	1	0	7	0	0	0	10
8	2	0	0	0	0	1	0	3
Others	0	0	0	0	1	6	0	7
Total	34	5	13	48	2	17	1	120

### The SNP of *SNQ2* was associated with the SNP of the sequence type CTRG_05978, and their combination effectively distinguished the Clade 4 genotype

Since clade 4 was identified as the primary azole-resistant genotype (Yang et al. 2005, 2008, 2013, Zhou et al. 2016, 2022), we embarked on an additional investigation to determine the potential involvement of gene(s) near CTRG_05978 associated with drug resistance. Our study uncovered a missense mutation I993V due to the SNP (A2977G) in *SNQ2*, which is situated 25 kb upstream from CTRG_05978 and has been linked to drug susceptibility in *N. glabratus* (*C. glabrata*) and *C. auris* (Torelli et al. 2008, Wasi et al. 2019). This finding suggests a possible connection between the mutation in *SNQ2* and the sequence profile of CTRG_05978 (Table 2 and Table S3).

**Table 2.** The association of genotype and SNP of *SNQ2* at 2977 among 120 *C. tropicalis* isolates.

	Sequence of *SNQ2* at 2977 position			
Clade	A/A	A/G	G/G	Total
1	1	3	0	4
2	12	0	0	12
3	13	2	0	15
4	2	35	1	38
5	19	0	0	19
6	12	0	0	12
7	10	0	0	10
8	1	2	0	3
Others	7	0	0	7
Total	77	42	1	120

We observed the *SNQ2* A 2977 G had a high association with the genotype IV of CTRG_05978 (35/43 versus 13/77, *P* < .001, at phi = 0.631) and the clade 4 genotype (36/43 versus 2/77, *P* < .001, at phi = 0.820). The combination of the *SNQ2* A2977G and the genotype IV of CTRG_05978 appeared to be effective in distinguishing the clade 4 strains (35/35 versus 3/85, *P* < .001), and the relationship exhibited a notably high association (phi = 0.943).

To obtain a more comprehensive understanding of whether the SNPs of *SNQ2* and CTRG_5978 could efficiently identify the clade 4 in *C. tropicalis*, we extended a thorough analysis of whole-genome data from 161 *C. tropicalis* strains available in the NCBI database (https://www.ncbi.nlm.nih.gov/sra). These strains encompass geographic variations, with the majority originating from Asia (76%), followed by Oceania (15%), Europe (5%), and North America (3%) (Table S5).

Overall, our analysis revealed a significant prevalence of the *SNQ2* A2977G and CTRG_05978 genotype IV among the clade 4 strains (Table 3). These findings demonstrated that *SNQ2* and CTRG_05978, indeed, could serve as markers for identifying clade 4 *C. tropicalis* (95/96 versus 22/185, *P* < .001, at phi = 0.838).

**Table 3.** The association of genotype and SNPs of *SNQ2* and CTRG_05978 among 281 *C. tropicalis* isolates.

		CTRG_05978		*SNQ2* at 2977 position		Type IV of CRTG_05978 and *SNQ2* G2977	
Clade		Type IV (ATRA)	Others	G	A	Y	N
1	Taiwan	0	4	3	1	0	4
	Worldwide	0	1	0	1	0	1
	Subtotal	0	5	3	2	0	5
2	Taiwan	0	12	0	12	0	12
	Worldwide	0	16	0	16	0	16
	Subtotal	0	28	0	28	0	28
3	Taiwan	3	12	2	13	0	15
	Worldwide	0	13	0	13	0	13
	Subtotal	3	25	2	26	0	28
4	Taiwan	36	2	36	2	35	3
	Worldwide	60	19	69	10	60	19
	Subtotal	96	21	105	12	95	22
5	Taiwan	0	19	0	19	0	19
	Worldwide	0	15	0	15	0	15
	Subtotal	0	34	0	34	0	34
6	Taiwan	2	10	0	12	0	12
	Worldwide	0	16	0	16	0	16
	Subtotal	2	26	0	28	0	28
7	Taiwan	7	3	0	10	0	10
	Worldwide	0	4	1	3	0	4
	Subtotal	7	7	1	13	0	14
8	Taiwan	0	3	2	1	0	3
	Worldwide	0	2	2	0	0	2
	Subtotal	0	5	4	1	0	5
Others	Taiwan	0	7	0	7	0	7
	Worldwide	11	4	3	12	1	14
	Subtotal	11	11	3	19	1	21
Total		119	162	118	163	96	185

### TaqMan SNP genotyping assay was useful in identifying CTRG_05978 type IV and *SNQ2* A2977G

To identify predominant azole-resistant clade 4 *C. tropicalis* strains rapidly, we established a TaqMan SNP Genotyping Assay for detecting the genotype of CTRG_05978 type IV and *SNQ2* A2977G. The specificity of the primers and probes was tested using purified genomic DNAs from 18 different *C. tropicalis* strains including ATCC 750 and 17 strains from clinical settings (Fig. S2A).

For the CTRG_05978 genotype, all type IV strains (YM140156, YM180233, YM180351, YM180473, YM180681, and YM180969) were located in the heterozygous area (green spots), while the others were located in homozygous areas (red and blue spots) (Fig. S2B). In the case of the *SNQ2* A2977G assay, it also specifically detected four mutation strains (blue and green spots) (Fig. S2C). Overall, the results of TaqMan SNP Genotyping Assay were consistent with Sanger sequencing or Whole Genome Sequencing, suggesting that it was a rapid and convenient method for identifying the predominant azole-resistant clade 4 *C. tropicalis* genotype through detecting SNPs of CTRG_05978 and *SNQ2*.

## Discussion

The *C. tropicalis* MLST scheme was established in 2005, prior to our awareness of the duplicated *XYR1* genes within the *C. tropicalis* genome. MLST has been used globally to characterize genetic diversity, phylogenetic population structure, and the epidemiology of *C. tropicalis* strains (Tavanti et al. 2005). Currently, there are more than 800 DSTs among a total of 1704 (as of 18 March 2024). The effect of these two gene copies on the discriminatory power of the MLST analysis needs to be thoroughly investigated.

Mutations serve as the foundational material for evolution. While it is conceivable that *C. tropicalis* might not require two functional *XYR1* genes to the same extent as its ancestor, hence driving adaptive mutations, it is challenging to attribute the mutation's linkage to the MLST cluster and its relevance to drug resistance and virulence solely to genetic drift and natural selection. Intriguingly, *SNQ2* is near CTRG_05978 and is implicated in azole susceptibility. The disruption of *CgSNQ2* led to decreased azole resistance in *N. glabratus* (*C. glabrata*) (Torelli et al. 2008), and *SNQ2* showed upregulated expression in azole-resistant clinical *C. auris* strains (Wasi et al. 2019). Furthermore, based on the predicted structure (https://www.uniprot.org/), the 993 amino acid position of Snq2 located within ATP-binding cassette transport domain, may potentially play a role in azole resistance in *C. tropicalis*. Therefore, genotypes of *SNQ2* A2977G and CTRG_05978 type IV tend to be linked with each other in clade 4 of *C. tropicalis* (95/117). This may be ascribed the short physical distance (∼25 Kb) between the two loci and/or hitchhiking effect under drug-related selective pressure. However, the linkage is not perfect. This is understandable because the frequency of the hypothesized advantageous mutation may be inching towards unity. The discordance may result from incomplete fixation. Hence, *C. tropicalis* can be in the presence or absence of drug depending on its surrounding. Furthermore, it has been reported that overexpression of the mutated azole target gene *ERG11* is the major mechanism contributing to azole resistance in clade 4 strains (Zhou et al. 2022, Fan et al. 2023). Consequently, the *SNQ2* A2977G alteration may not have caused a sufficiently strong selective sweep to arrive at complete fixation. In addition, independent mutations may also explain the limited selective advantage conveyed by *SNQ2* A2977G. Moreover, whether the *SNQ2* A2977G alteration resulting in amino acid I993V change has complementary impacts on the azole resistance of *C. tropicalis* needs further investigation.

Despite the lack of comprehensive information, a method for detecting the potential marker for rapidly identifying the azole-resistant *C. tropicalis* with the clade 4 genotype has been developed and tested in the present study. The potential markers involve only a few nucleotide variations, which can be detected using designed capture probes targeting the SNPs of Snq2 I993V and the first 4-mer of CTRG_05978. Even though techniques such as real-time PCR applications, ELISA-like assays, or microarray analysis may also be suitable for the purpose, an easily accessible and convenient method for detection as our findings are more practical for clinical settings. In this study, we presented the TaqMan SNP genotype assay as a rapid method for identifying these SNPs in CTRG_05978 and the *SNQ2* A2977G.

We detected that some *C. tropicalis* isolates of clade 4 did not belong to the CTRG_05987 type IV cluster and/or do not carry the *SNQ2* A2977G mutation and one nonclade 4 strain belonged to the CTRG_05987 type IV cluster and carried the *SNQ2* A2977G mutation. To increase the accuracy of rapidly identifying azole-resistant clade 4 *C. tropicalis*, we suggest determining whether the strains carry *ERG11* mutations in addition to the genotypes of CTRG_05987 and *SNQ2* identified in the present study.

## Conclusion

Recent studies have highlighted that *C. tropicalis* not only occurs in clinical settings but also inhabits various environmental niches and forms associations, including plant surfaces, soil, and commensal relationships with humans (Berger et al. 2010, Lo et al. 2017, Hirayama et al. 2020). The potential markers involve only a few nucleotide variations, which can be detected using designed capture probes targeting the SNPs of *SNQ2* A2977G and the first 4-mer, ATRA, of CTRG_05978. Our findings can greatly contribute to the development of targeted diagnostics resulting in a proper prescription for therapy. In conclusion, our study presented a promising approach for monitoring and managing the spread of clade 4 *C. tropicalis*, thereby offering a potential tool for mitigating significant health risks for vulnerable patient populations.

## Supplementary Material

foae025_Supplemental_Files

## Sequence information

The sequences will be made available under Supplementary data.
