# Supplementary material for: Rapid identification of the predominant azole-resistant genotype in Candida tropicalis
Source: FEMS Yeast Res. 2024 Oct 21;24:foae025. doi: 10.1093/femsyr/foae025 (PMC11500656; doi:10.1093/femsyr/foae025)
Supplement: foae025_Supplemental_Files [file foae025_supplemental_files.zip › FEMSYR Supplementary Figure20240801.docx]

**Supplementary Figure**


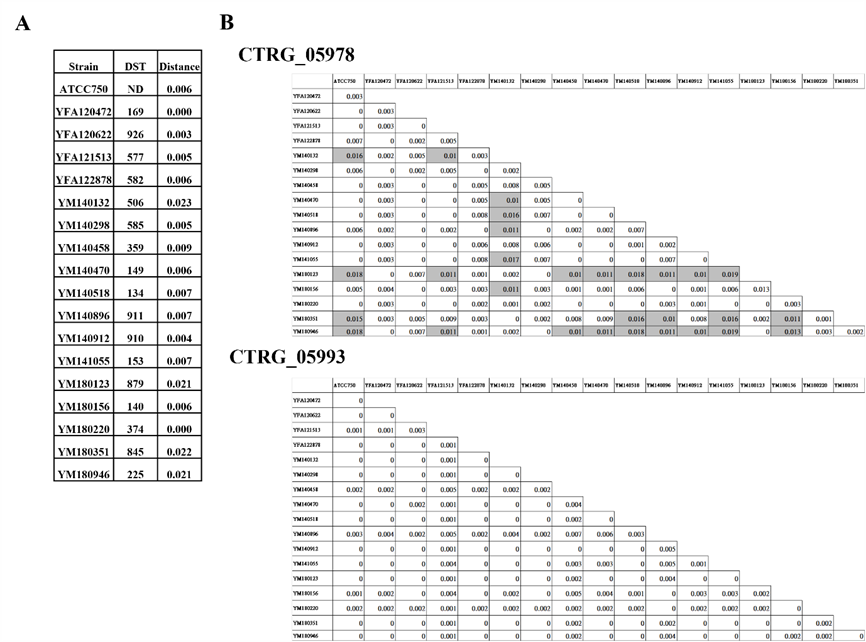


Figure S1. The distance of intra and inter-genotype for two *XYR1* genes. (A) The pairwise distance of two *XYR1* within the same strain. (B) The pairwise distance of CTRG_05978 and CTRG_05993 between the two strains. (Gray ≥ 0.01)


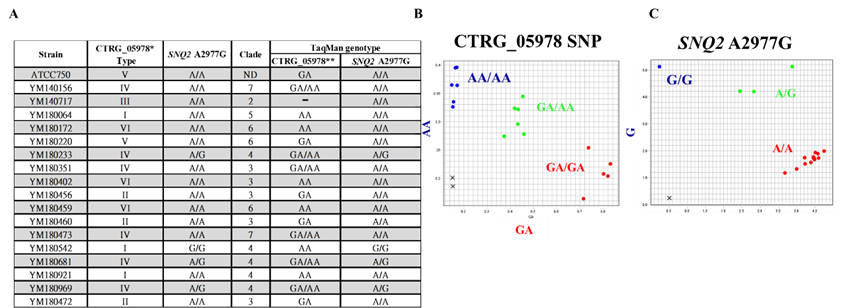


Figure S2. TaqMan Genotyping Assay enables rapid detection of the genotype IV of CTRG_05978 and the *SNQ2* A2977G. (A) TaqMan Genotyping Assay was performed on 18 *C. tropicalis* strains, including 17 clinical strains, and ATCC750, with diverse CTRG_05978 and *SNQ2* genotypes (B) Allele discrimination plot for CTRG_05978 displaying alleles as GA/GA homozygous (lower right red cluster), GA/AA heterozygous (middle green cluster), and AA/AA homozygous (upper left blue cluster). The middle green cluster strains correspond to the CTRG_05978 type IV genotype (GA/AA), and there are 6 strains located in the middle green cluster, including YM140156, YM180233, YM180351, YM180473, YM180681, and YM180969. (C) Allele discrimination plot for *SNQ2* A2977G, presenting alleles as G/G homozygous (lower right red cluster), G/A heterozygous (middle green cluster), and A/A homozygous (upper left blue cluster). Four strains exhibited mutations in *SNQ2* mutation. By combining the results of CTRG_05978 and *SNQ2* genotypes using the TaqMan Genotyping Assay, the identification of CTRG_05978 type IV and *SNQ2* A2977G becomes straightforward.

*Based on the first 4mers of CTRG_05978, we can define 7 types in those strains.

Type I is ATAA; type II is ATGA; type III is ATGT; type IV is ATRA; type V is ATGW, type VI is ATRW, and type VII is ATWA. **CTRG_5979 detected GA and AA SNP.
