# Supplementary material for: Rapid identification of the predominant azole-resistant genotype in Candida tropicalis
Source: FEMS Yeast Res. 2024 Oct 21;24:foae025. doi: 10.1093/femsyr/foae025 (PMC11500656; doi:10.1093/femsyr/foae025)
Supplement: foae025_Supplemental_Files [file foae025_supplemental_files.zip › FEMSYR Supplementary Table202400801.docx]

Table S1

Primers used in the present study.

| **Name** | **Genes** | **Sequences** | **Position*** | **Reference** |
| --- | --- | --- | --- | --- |
| **MLST** | | | | |
| HJL2796 | *ICL1-F* | CAACAGATTGGTTGCCATCAGAGC | +675 – +698 | (Tavanti *et al.* 2005) |
| HJL2797 | *ICL1-R* | CGAAGTCATCAACAGCCAAAGCAG | +1412 – +1435 | (Tavanti *et al.* 2005) |
| HJL2799 | *MDR1-F* | GGCTGGAGATGGACTTTTTGG | +772 – +792 | (Zhou *et al.* 2022) |
| HJL2800 | *MDR1-R* | TGGAGCACCAAACAATGGG | +1491 – +1509 | (Zhou *et al.* 2022) |
| HJL2801 | *SAPT2-F* | CACAGAAACGCCTCCGTTTTG | -46 – -26 | (Zhou *et al.* 2022) |
| HJL2803 | *SAPT2-R* | CACTGGTAGCTGAAGGAG | +728 – +745 | (Zhou *et al.* 2022) |
| HJL2804 | *SAPT4-F* | TACCCATTGGAATGTATTGTTGA | -103 – -81 | (Zhou *et al.* 2022) |
| HJL2805 | *SAPT4-R* | CCAGTTGTTGAATCTGATGAATT | +637 – +659 | (Zhou *et al.* 2022) |
| HJL2807 | *XYR1-F* | CCAACAACTATAAAGAGATCGACA | -84 – -61 | (Zhou *et al.* 2022) |
| HJL2808 | *XYR1-R* | GCAAGTATGGGTGATGTTCAAT | +580 – +601 | (Zhou *et al.* 2022) |
| HJL2811 | *ZWF1a-F* | GTCWTATGATTCMTTYGGWGA | +3 – +23 | (Zhou *et al.* 2022) |
| HJL2812 | *ZWF1a-R* | GARACTGGTCTTTCCATRGT | +751 – +770 | (Zhou *et al.* 2022) |
| HJL3097 | *CTRG_05978-F* | GACGATTACGGTCACAGAATC | -172 – -152 | this study |
| HJL3098 | *CTRG_05993-F* | CTAATAGCAAGTTATTGCCCC | -89 – -69 | this study |
| HJL3174 | *SNQ2-F* | TGCTCTTGGTGGATCTACTG | +2523 – +2542 | this study |
| HJL3175 | *SNQ2-R* | CTTGATCTATCACCAATATCACC | +3253 – +3275 | this study |
| TaqMan SNP genotyping assay | | | | |
| CTRG_05978 Type IV | Forward Primer | CGTCATATACTCGGAAGGACTTTCTCTA | -62 – -33 | this study |
|  | Reverse Primer | CGATCTCTTTATAGTTGTTGGAGAAGTGA | +13 – +42 | this study |
|  | Probe 1 (ATGA) | CCCCTTAAATAATGATTAAAT | -11 – +10 | this study |
|  | Probe 2 (ATAA) | CCCCTTAAATAATAATTAAAT | -11 – +10 | this study |
| *SNQ2* A2977G | Forward Primer | TGAAAAGATCATTGATGTTTTGGATATGAAAGG | +2931 – +2963 | this study |
|  | Reverse Primer | CGTTGCTCGACATTTAAACCATTACC | +2992 – +3017 | this study |
|  | Probe 1 (A) | CCGATGCCATTGTTG | +2969 – +2983 | this study |
|  | Probe 2 (G) | CCGATGCCGTTGTTG | +2969 – +2983 | this study |

*Position of translation initiation site of ATG as +1

Table S2 The sequence of the first 4-mer of CTRG_05978 and CTRG_05993

| CTRG_05978 Type | Clade | DST | Strain | The first 4-mer of CTRG_05978 | The first 4-mer of CTRG_05993 |
| --- | --- | --- | --- | --- | --- |
| I | 5 | 140 | YM180156 | ATAA | ATGT |
| II | 3 | 872 | YM180490 | ATGA | ATGT |
| III | 2 | 153 | YM141055 | ATGT | ATGT |
| IV | 4 | 225 | YFA120301 | ATRA | ATGT |
| V | 6 | 374 | NC1S04 | ATGW | ATGT |
| VI | 5 | 910 | YM140912 | ATRW | ATGT |
| VII | 3 | 370 | YM140797 | ATWA | ATGT |

Table S3 SNPs of CTRG_05978 and *SNQ2*

| **CTRG_05978 Type** | ***SNQ2* (A2977G)** | **Clade** | **DST** | **Strain** | **The first of 60-mer of CTRG_05978** | **Reference** |
| --- | --- | --- | --- | --- | --- | --- |
| **I ( ATAA)** | **A/A** | **1** | **190** | **YM060500** | **ATAATTAAATTTTTCACTTCTCCAACAACTATAAAGAGATCGACATTTCTCTTCAATTAT** | **(Li *et al.* 2009)** |
| **I ( ATAA)** | **A/G** | **1** | **196** | **YM060342** | **ATAATKWWWWWWTTYWYYWCTYCWMCAACWAYWAWRARRWSRWCRWYWYTYYTCWWYWAT** | **(Li *et al.* 2009)** |
| **I ( ATAA)** | **A/G** | **1** | **582** | **YM140516** | **ATAATTAAATTTTTCACTTCTCCAACAACTATAAAGAGATCGACATTTCTCTTCAATTAT** | **This study** |
| **I ( ATAA)** | **A/G** | **1** | **582** | **YM140682** | **ATAATTAAATTTTTCACTTCTCCAACAACTATAAAGAGATCGACATTTCTCTTCAATTAT** | **This study** |
| **I ( ATAA)** | **A/G** | **3** | **585** | **YM140298** | **ATAATTAAATTTTTCACTTCTCCAACAACTATAAAGAGATCGACATTTCTCTTCAATTAT** | **(Tseng *et al.* 2022)** |
| **I ( ATAA)** | **A/A** | **3** | **965** | **YM140465** | **ATAATTAAATTTTTCACTTCTCCAACAACTATAAAGAGATCGACATTTCTCTTCAATTAT** | **This study** |
| **I ( ATAA)** | **A/A** | **3** | **965** | **YM180451** | **ATAATTAAATTTTTCACTTCTCCAACAACTATAAAGAGATCGACATTTCTCTTCAATTAT** | **This study** |
| **I ( ATAA)** | **A/A** | **3** | **965** | **YM180453** | **ATAATTAAATTTTTCACTTCTCCAACAACTATAAAGAGATCGACATTTCTCTTCAATTAT** | **This study** |
| **I ( ATAA)** | **A/G** | **3** | **971** | **YM180043** | **ATAATKWWWWWWTTYWYYWCTYCWMCAACWAYWAWRARRWSRWCRWYWYTYYTCWWYWAT** | **This study** |
| **I ( ATAA)** | **G/G** | **4** | **546** | **YM180542** | **ATAATTAAATTTTTCACTTCTCCAACAACTATAAAGAGATCGACATTTCTCTTCAATTAT** | **This study** |
| **I ( ATAA)** | **A/A** | **4** | **508** | **YM180921** | **ATAATTAAATTTTTCACTTCTCCAACAACTATAAAGAGATCGACATTTCTCTTCAATTAT** | **This study** |
| **I ( ATAA)** | **A/A** | **5** | **98** | **YM180064** | **ATAATTAAATTTTTCACTTCTCCAACAACTATAAAGAGATCGACATTTCTCTTCAATTAT** | **This study** |
| **I ( ATAA)** | **A/A** | **5** | **98** | **YM181190** | **ATAATTAAATTTTTCACTTCTCCAACAACTATAAAGAGATCGACATTTCTCTTCAATTAT** | **This study** |
| **I ( ATAA)** | **A/A** | **5** | **140** | **YM060098** | **ATAATTAAATTTTTCACTTCTCCAACAACTATAAAGAGATCGACATTTCTCTTCAATTAT** | **(Li *et al.* 2009)** |
| **I ( ATAA)** | **A/A** | **5** | **140** | **YM060828** | **ATAATTAAATTTTTCACTTCTCCAACAACTATAAAGAGATCGACATTTCTCTTCAATTAT** | **(Li *et al.* 2009)** |
| **I ( ATAA)** | **A/A** | **5** | **140** | **YM140225** | **ATAATTAAATTTTTCACTTCTCCAACAACTATAAAGAGATCGACATTTCTCTTCAATTAT** | **(Tseng *et al.* 2022)** |
| **I ( ATAA)** | **A/A** | **5** | **140** | **YM140977** | **ATAATTAAATTTTTCACTTCTCCAACAACTATAAAGAGATCGACATTTCTCTTCAATTAT** | **(Tseng *et al.* 2022)** |
| **I ( ATAA)** | **A/A** | **5** | **140** | **YM141125** | **ATAATTAAATTTTTCACTTCTCCAACAACTATAAAGAGATCGACATTTCTCTTCAATTAT** | **This study** |
| **I ( ATAA)** | **A/A** | **5** | **140** | **YM180054** | **ATAATTAAATTTTTCACTTCTCCAACAACTATAAAGAGATCGACATTTCTCTTCAATTAT** | **This study** |
| **I ( ATAA)** | **A/A** | **5** | **140** | **YM180156** | **ATAATTAAATTTTTCACTTCTCCAACAACTATAAAGAGATCGACATTTCTCTTCAATTAT** | **(Tseng *et al.* 2022)** |
| **I ( ATAA)** | **A/A** | **5** | **140** | **YM180483** | **ATAATTAAATTTTTCACTTCTCCAACAACTATAAAGAGATCGACATTTCTCTTCAATTAT** | **(Tseng *et al.* 2022)** |
| **I ( ATAA)** | **A/A** | **5** | **140** | **YM180486** | **ATAATTAAATTTTTCACTTCTCCAACAACTATAAAGAGATCGACATTTCTCTTCAATTAT** | **This study** |
| **I ( ATAA)** | **A/A** | **5** | **140** | **YM180942** | **ATAATTAAATTTTTCACTTCTCCAACAACTATAAAGAGATCGACATTTCTCTTCAATTAT** | **This study** |
| **I ( ATAA)** | **A/A** | **5** | **140** | **YM990275** | **ATAATTAAATTTTTCACTTCTCCAACAACTATAAAGAGATCGACATTTCTCTTCAATTAT** | **(Chou *et al.* 2007)** |
| **I ( ATAA)** | **A/A** | **5** | **140** | **YM990592** | **ATAATTAAATTTTTCACTTCTCCAACAACTATAAAGAGATCGACATTTCTCTTCAATTAT** | **(Chou *et al.* 2007)** |
| **I ( ATAA)** | **A/A** | **5** | **1142** | **YM990659** | **ATAATTAAATTTTTCACTTCTCCAACAACTATAAAGAGATCGACATTTCTCTTCAATTAT** | **(Chou *et al.* 2007)** |
| **I ( ATAA)** | **A/A** | **5** | **911** | **YM140479** | **ATAATTAAATTTTTCACTTCTCCAACAACTATAAAGAGATCGACATTTCTCTTCAATTAT** | **This study** |
| **I ( ATAA)** | **A/A** | **5** | **911** | **YM140896** | **ATAATTAAATTTTTCACTTCTCCAACAACTATAAAGAGATCGACATTTCTCTTCAATTAT** | **(Tseng *et al.* 2022)** |
| **I ( ATAA)** | **A/A** | **5** | **953** | **YM180956** | **ATAATTAAATTTTTCACTTCTCCAACAACTATAAAGAGATCGACATTTCTCTTCAATTAT** | **This study** |
| **I ( ATAA)** | **A/A** | **6** | **197** | **YM060109** | **ATAATKWWWWWWTTYWYYWCTYCWMCAACWAYWAWRARRWSRWCRWYWYTYYTCWWYWAT** | **(Li *et al.* 2009)** |
| **I ( ATAA)** | **A/A** | **7** | **138** | **YM990236** | **ATAATTAAATTTTTCACTTCTCCAACAACTATAAAGAGATCGACATTTCTCTTCAATTAT** | **(Chou *et al.* 2007)** |
| **I ( ATAA)** | **A/A** | **7** | **666** | **YM140303** | **ATAATTAAATTTTTCACTTCTCCAACAACTATAAAGAGATCGACATTTCTCTTCAATTAT** | **This study** |
| **I ( ATAA)** | **A/G** | **8** | **142** | **YM990329** | **ATAATKWWWWWWTTYWYYWCTYCWMCAACWAYWAWRARRWSRWCRWYWYTYYTCWWYWAT** | **(Chou *et al.* 2007)** |
| **I ( ATAA)** | **A/G** | **8** | **195** | **YM060051** | **ATAATKWWWWWWTTYWYYWCTYCWMCAACWAYWAWRARRWSRWCRWYWYTYYTCWWYWAT** | **(Li *et al.* 2009)** |
| **II (ATGA)** | **A/A** | **3** | **872** | **YM180456** | **ATGATTAAATTTTTCACTTCTCCAACAACTATAAAGAGATCGACATTTCTCTTCAATTAT** | **This study** |
| **II (ATGA)** | **A/A** | **3** | **872** | **YM180460** | **ATGATTAAATTTTTCACTTCTCCAACAACTATAAAGAGATCGACATTTCTCTTCAATTAT** | **This study** |
| **II (ATGA)** | **A/A** | **3** | **872** | **YM180472** | **ATGATTAAATTTTTCACTTCTCCAACAACTATAAAGAGATCGACATTTCTCTTCAATTAT** | **This study** |
| **II (ATGA)** | **A/A** | **3** | **872** | **YM180490** | **ATGATTAAATTTTTCACTTCTCCAACAACTATAAAGAGATCGACATTTCTCTTCAATTAT** | **This study** |
| **II (ATGA)** | **A/A** | **7** | **171** | **YM060172** | **ATGATTAAATTTTTCACTTCTCCAACAACTATAAAGAGATCGACATTTCTCTTCAATTAT** | **This study** |
| **III (ATGT)** | **A/A** | **2** | **27** | **YM060481** | **ATGTTTAAATTTTTCACTTCTCCAACAACTATAAAGAGATCGACATTTCTCTTCAATTAT** | **This study** |
| **III (ATGT)** | **A/A** | **2** | **90** | **YM140365** | **ATGTTTAAATTTTTCACTTCTCCAACAACTATAAAGAGATCGACATTTCTCTTCAATTAT** | **This study** |
| **III (ATGT)** | **A/A** | **2** | **134** | **YM060299** | **ATGTTTAAATTTTTCACTTCTCCAACAACTATAAAGAGATCGACATTTCTCTTCAATTAT** | **(Li *et al.* 2009)** |
| **III (ATGT)** | **A/A** | **2** | **134** | **YM060507** | **ATGTTTAAATTTTTCACTTCTCCAACAACTATAAAGAGATCGACATTTCTCTTCAATTAT** | **(Li *et al.* 2009)** |
| **III (ATGT)** | **A/A** | **2** | **134** | **YM060508** | **ATGTTTAAATTTTTCACTTCTCCAACAACTATAAAGAGATCGACATTTCTCTTCAATTAT** | **(Li *et al.* 2009)** |
| **III (ATGT)** | **A/A** | **2** | **134** | **YM060512** | **ATGTTTAAATTTTTCACTTCTCCAACAACTATAAAGAGATCGACATTTCTCTTCAATTAT** | **(Li *et al.* 2009)** |
| **III (ATGT)** | **A/A** | **2** | **134** | **YM140518** | **ATGTTTAAATTTTTCACTTCTCCAACAACTATAAAGAGATCGACATTTCTCTTCAATTAT** | **(Tseng *et al.* 2022)** |
| **III (ATGT)** | **A/A** | **2** | **134** | **YM140717** | **ATGTTTAAATTTTTCACTTCTCCAACAACTATAAAGAGATCGACATTTCTCTTCAATTAT** | **This study** |
| **III (ATGT)** | **A/A** | **2** | **153** | **YM141055** | **ATGTTTAAATTTTTCACTTCTCCAACAACTATAAAGAGATCGACATTTCTCTTCAATTAT** | **(Tseng *et al.* 2022)** |
| **III (ATGT)** | **A/A** | **2** | **200** | **YM060379** | **ATGTTTAAATTTTTCACTTCTCCAACAACTATAAAGAGATCGACATTTCTCTTCAATTAT** | **(Li *et al.* 2009)** |
| **III (ATGT)** | **A/A** | **2** | **200** | **YM060800** | **ATGTTTAAATTTTTCACTTCTCCAACAACTATAAAGAGATCGACATTTCTCTTCAATTAT** | **(Li *et al.* 2009)** |
| **III (ATGT)** | **A/A** | **2** | **202** | **YM060565** | **ATGTTTAAATTTTTCACTTCTCCAACAACTATAAAGAGATCGACATTTCTCTTCAATTAT** | **(Li *et al.* 2009)** |
| **III (ATGT)** | **A/A** | **6** | **149** | **YM140470** | **ATGTTTAAATTTTTCACTTCTCCAACAACTATAAAGAGATCGACATTTCTCTTCAATTAT** | **(Tseng *et al.* 2022)** |
| **IV (ATRA)** | **A/A** | **3** | **189** | **YM060383** | **ATRATWAWWWWWTTYWYYWCTYCWMCAACWAYWAWRARRWSRWCRWYWYTYYTCWWYWAT** | **(Li *et al.* 2009)** |
| **IV (ATRA)** | **A/A** | **3** | **845** | **YM141035** | **ATRATTAAATTTTTCACTTCTCCAACAACTATAAAGAGATCGACATTTCTCTTCAATTAT** | **This study** |
| **IV (ATRA)** | **A/A** | **3** | **845** | **YM180351** | **ATRATTAAATTTTTCACTTCTCCAACAACTATAAAGAGATCGACATTTCTCTTCAATTAT** | **(Tseng *et al.* 2022)** |
| **IV (ATRA)** | **A/G** | **4** | **225** | **YM140093** | **ATRATTAAATTTTTCACTTCTCCAACAACTATAAAGAGATCGACATTTCTCTTCAATTAT** | **This study** |
| **IV (ATRA)** | **A/G** | **4** | **225** | **YM140168** | **ATRATTAAATTTTTCACTTCTCCAACAACTATAAAGAGATCGACATTTCTCTTCAATTAT** | **This study** |
| **IV (ATRA)** | **A/G** | **4** | **225** | **YM140219** | **ATRATTAAATTTTTCACTTCTCCAACAACTATAAAGAGATCGACATTTCTCTTCAATTAT** | **This study** |
| **IV (ATRA)** | **A/G** | **4** | **225** | **YM140372** | **ATRATTAAATTTTTCACTTCTCCAACAACTATAAAGAGATCGACATTTCTCTTCAATTAT** | **(Tseng *et al.* 2022)** |
| **IV (ATRA)** | **A/G** | **4** | **225** | **YM140443** | **ATRATTAAATTTTTCACTTCTCCAACAACTATAAAGAGATCGACATTTCTCTTCAATTAT** | **This study** |
| **IV (ATRA)** | **A/G** | **4** | **225** | **YM140444** | **ATRATTAAATTTTTCACTTCTCCAACAACTATAAAGAGATCGACATTTCTCTTCAATTAT** | **This study** |
| **IV (ATRA)** | **A/G** | **4** | **225** | **YM140449** | **ATRATTAAATTTTTCACTTCTCCAACAACTATAAAGAGATCGACATTTCTCTTCAATTAT** | **This study** |
| **IV (ATRA)** | **A/G** | **4** | **225** | **YM140453** | **ATRATTAAATTTTTCACTTCTCCAACAACTATAAAGAGATCGACATTTCTCTTCAATTAT** | **This study** |
| **IV (ATRA)** | **A/G** | **4** | **225** | **YM140454** | **ATRATTAAATTTTTCACTTCTCCAACAACTATAAAGAGATCGACATTTCTCTTCAATTAT** | **This study** |
| **IV (ATRA)** | **A/G** | **4** | **225** | **YM140586** | **ATRATTAAATTTTTCACTTCTCCAACAACTATAAAGAGATCGACATTTCTCTTCAATTAT** | **(Tseng *et al.* 2022)** |
| **IV (ATRA)** | **A/G** | **4** | **225** | **YM140779** | **ATRATTAAATTTTTCACTTCTCCAACAACTATAAAGAGATCGACATTTCTCTTCAATTAT** | **This study** |
| **IV (ATRA)** | **A/G** | **4** | **225** | **YM140935** | **ATRATTAAATTTTTCACTTCTCCAACAACTATAAAGAGATCGACATTTCTCTTCAATTAT** | **This study** |
| **IV (ATRA)** | **A/G** | **4** | **225** | **YM140950** | **ATRATTAAATTTTTCACTTCTCCAACAACTATAAAGAGATCGACATTTCTCTTCAATTAT** | **This study** |
| **IV (ATRA)** | **A/G** | **4** | **225** | **YM140954** | **ATRATTAAATTTTTCACTTCTCCAACAACTATAAAGAGATCGACATTTCTCTTCAATTAT** | **This study** |
| **IV (ATRA)** | **A/G** | **4** | **225** | **YM140969** | **ATRATTAAATTTTTCACTTCTCCAACAACTATAAAGAGATCGACATTTCTCTTCAATTAT** | **This study** |
| **IV (ATRA)** | **A/G** | **4** | **225** | **YM141153** | **ATRATTAAATTTTTCACTTCTCCAACAACTATAAAGAGATCGACATTTCTCTTCAATTAT** | **(Tseng *et al.* 2022)** |
| **IV (ATRA)** | **A/G** | **4** | **225** | **YM180235** | **ATRATTAAATTTTTCACTTCTCCAACAACTATAAAGAGATCGACATTTCTCTTCAATTAT** | **(Tseng *et al.* 2022)** |
| **IV (ATRA)** | **A/G** | **4** | **225** | **YM180946** | **ATRATTAAATTTTTCACTTCTCCAACAACTATAAAGAGATCGACATTTCTCTTCAATTAT** | **(Tseng *et al.* 2022)** |
| **IV (ATRA)** | **A/G** | **4** | **225** | **YM180969** | **ATRATTAAATTTTTCACTTCTCCAACAACTATAAAGAGATCGACATTTCTCTTCAATTAT** | **(Tseng *et al.* 2022)** |
| **IV (ATRA)** | **A/G** | **4** | **506** | **YM140066** | **ATRATTAAATTTTTCACTTCTCCAACAACTATAAAGAGATCGACATTTCTCTTCAATTAT** | **(Tseng *et al.* 2022)** |
| **IV (ATRA)** | **A/G** | **4** | **506** | **YM140132** | **ATRATTAAATTTTTCACTTCTCCAACAACTATAAAGAGATCGACATTTCTCTTCAATTAT** | **(Tseng *et al.* 2022)** |
| **IV (ATRA)** | **A/G** | **4** | **506** | **YM140258** | **ATRATTAAATTTTTCACTTCTCCAACAACTATAAAGAGATCGACATTTCTCTTCAATTAT** | **(Tseng *et al.* 2022)** |
| **IV (ATRA)** | **A/G** | **4** | **506** | **YM140441** | **ATRATTAAATTTTTCACTTCTCCAACAACTATAAAGAGATCGACATTTCTCTTCAATTAT** | **This study** |
| **IV (ATRA)** | **A/G** | **4** | **506** | **YM180681** | **ATRATTAAATTTTTCACTTCTCCAACAACTATAAAGAGATCGACATTTCTCTTCAATTAT** | **(Tseng *et al.* 2022)** |
| **IV (ATRA)** | **A/G** | **4** | **506** | **YM180837** | **ATRATTAAATTTTTCACTTCTCCAACAACTATAAAGAGATCGACATTTCTCTTCAATTAT** | **(Tseng *et al.* 2022)** |
| **IV (ATRA)** | **A/G** | **4** | **506** | **YM180950** | **ATRATTAAATTTTTCACTTCTCCAACAACTATAAAGAGATCGACATTTCTCTTCAATTAT** | **(Tseng *et al.* 2022)** |
| **IV (ATRA)** | **A/G** | **4** | **506** | **YM180978** | **ATRATTAAATTTTTCACTTCTCCAACAACTATAAAGAGATCGACATTTCTCTTCAATTAT** | **(Tseng *et al.* 2022)** |
| **IV (ATRA)** | **A/G** | **4** | **506** | **YM181042** | **ATRATTAAATTTTTCACTTCTCCAACAACTATAAAGAGATCGACATTTCTCTTCAATTAT** | **(Tseng *et al.* 2022)** |
| **IV (ATRA)** | **A/G** | **4** | **546** | **YM180538** | **ATRATTAAATTTTTCACTTCTCCAACAACTATAAAGAGATCGACATTTCTCTTCAATTAT** | **This study** |
| **IV (ATRA)** | **A/G** | **4** | **592** | **YM180233** | **ATRATTAAATTTTTCACTTCTCCAACAACTATAAAGAGATCGACATTTCTCTTCAATTAT** | **(Tseng *et al.* 2022)** |
| **IV (ATRA)** | **A/G** | **4** | **595** | **YM140907** | **ATRATTAAATTTTTCACTTCTCCAACAACTATAAAGAGATCGACATTTCTCTTCAATTAT** | **This study** |
| **IV (ATRA)** | **A/G** | **4** | **600** | **YM140789** | **ATRATTAAATTTTTCACTTCTCCAACAACTATAAAGAGATCGACATTTCTCTTCAATTAT** | **This study** |
| **IV (ATRA)** | **A/A** | **4** | **667** | **YM141031** | **ATRATTAAATTTTTCACTTCTCCAACAACTATAAAGAGATCGACATTTCTCTTCAATTAT** | **This study** |
| **IV (ATRA)** | **A/G** | **4** | **879** | **YM180123** | **ATRATTAAATTTTTCACTTCTCCAACAACTATAAAGAGATCGACATTTCTCTTCAATTAT** | **(Tseng *et al.* 2022)** |
| **IV (ATRA)** | **A/G** | **4** | **924** | **YM140982** | **ATRATTAAATTTTTCACTTCTCCAACAACTATAAAGAGATCGACATTTCTCTTCAATTAT** | **This study** |
| **IV (ATRA)** | **A/G** | **4** | **1096** | **YM181122** | **ATRATTAAATTTTTCACTTCTCCAACAACTATAAAGAGATCGACATTTCTCTTCAATTAT** | **This study** |
| **IV (ATRA)** | **A/A** | **6** | **921** | **YM140073** | **ATRATKWWWWWWTTYWYYWCTYCWMCAACWAYWAWRARRWSRWCRWYWYTYYTCWWYWAT** | **This study** |
| **IV (ATRA)** | **A/A** | **6** | **1095** | **YM181080** | **ATRATWAWWWWWTTYWYYWCTYCWMCAACWAYWAWRARRWSRWCRWYWYTYYTCWWYWAT** | **This study** |
| **IV (ATRA)** | **A/A** | **7** | **139** | **YM060369** | **ATRATTAAATTTTTCACTTCTCCAACAACTATAAAGAGATCGACATTTCTCTTCAATTAT** | **(Li *et al.* 2009)** |
| **IV (ATRA)** | **A/A** | **7** | **139** | **YM140156** | **ATRATTAAATTTTTCACTTCTCCAACAACTATAAAGAGATCGACATTTCTCTTCAATTAT** | **This study** |
| **IV (ATRA)** | **A/A** | **7** | **139** | **YM990268** | **ATRATTAAATTTTTCACTTCTCCAACAACTATAAAGAGATCGACATTTCTCTTCAATTAT** | **(Chou *et al.* 2007)** |
| **IV (ATRA)** | **A/A** | **7** | **152** | **YM990652** | **ATRATTAAATTTTTCACTTCTCCAACAACTATAAAGAGATCGACATTTCTCTTCAATTAT** | **(Chou *et al.* 2007)** |
| **IV (ATRA)** | **A/A** | **7** | **184** | **YM060210** | **ATRATTAAATTTTTCACTTCTCCAACAACTATAAAGAGATCGACATTTCTCTTCAATTAT** | **(Li *et al.* 2009)** |
| **IV (ATRA)** | **A/A** | **7** | **833** | **YM180473** | **ATRATTAAATTTTTCACTTCTCCAACAACTATAAAGAGATCGACATTTCTCTTCAATTAT** | **This study** |
| **IV (ATRA)** | **A/A** | **7** | **956** | **YM180522** | **ATRATTAAATTTTTCACTTCTCCAACAACTATAAAGAGATCGACATTTCTCTTCAATTAT** | **This study** |
| **V (ATGW)** | **A/A** | **6** | **374** | **YM180220** | **ATGWTTAAATTTTTCACTTCTCCAACAACTATAAAGAGATCGACATTTCTCTTCAATTAT** | **(Tseng *et al.* 2022)** |
| **V (ATGW)** | **A/A** | **ND** | **914** | **ATCC750** | **ATGWTTAAATTTTTCACTTCTCCAACAACTATAAAGAGATCGACATTTCTCTTCAATTAT** | **(Tseng *et al.* 2022)** |
| **VI (ATRW)** | **A/A** | **3** | **148** | **YM990574** | **ATRWTTAAATTTTTCACTTCTCCAACAACTATAAAGAGATCGACATTTCTCTTCAATTAT** | **(Chou *et al.* 2007)** |
| **VI (ATRW)** | **A/A** | **3** | **967** | **YM180402** | **ATRWTTAAATTTTTCACTTCTCCAACAACTATAAAGAGATCGACATTTCTCTTCAATTAT** | **This study** |
| **VI (ATRW)** | **A/A** | **5** | **910** | **YM140912** | **ATRWTTAAATTTTTCACTTCTCCAACAACTATAAAGAGATCGACATTTCTCTTCAATTAT** | **(Chou *et al.* 2007)** |
| **VI (ATRW)** | **A/A** | **6** | **149** | **YM060097** | **ATRWTTAAATTTTTCACTTCTCCAACAACTATAAAGAGATCGACATTTCTCTTCAATTAT** | **(Li *et al.* 2009)** |
| **VI (ATRW)** | **A/A** | **6** | **149** | **YM060177** | **ATRWTTAAATTTTTCACTTCTCCAACAACTATAAAGAGATCGACATTTCTCTTCAATTAT** | **(Li *et al.* 2009)** |
| **VI (ATRW)** | **A/A** | **6** | **149** | **YM140092** | **ATRWTTAAATTTTTCACTTCTCCAACAACTATAAAGAGATCGACATTTCTCTTCAATTAT** | **(Tseng *et al.* 2022)** |
| **VI (ATRW)** | **A/A** | **6** | **149** | **YM180172** | **ATRWTTAAATTTTTCACTTCTCCAACAACTATAAAGAGATCGACATTTCTCTTCAATTAT** | **This study** |
| **VI (ATRW)** | **A/A** | **6** | **421** | **YM180461** | **ATRWTTAAATTTTTCACTTCTCCAACAACTATAAAGAGATCGACATTTCTCTTCAATTAT** | **This study** |
| **VI (ATRW)** | **A/A** | **6** | **960** | **YM180459** | **ATRWTTAAATTTTTCACTTCTCCAACAACTATAAAGAGATCGACATTTCTCTTCAATTAT** | **This study** |
| **VI (ATRW)** | **A/A** | **6** | **1194** | **YM990579** | **ATRWTTAAATTTTTCACTTCTCCAACAACTATAAAGAGATCGACATTTCTCTTCAATTAT** | **(Chou *et al.* 2007)** |
| **VI (ATRW)** | **A/A** | **8** | **150** | **YM990593** | **ATRWTTAAATTTTTCACTTCTCCAACAACTATAAAGAGATCGACATTTCTCTTCAATTAT** | **(Chou *et al.* 2007)** |
| **VI (ATRW)** | **A/A** | **9** | **133** | **YM990136** | **ATRWTTAAATTTTTCACTTCTCCAACAACTATAAAGAGATCGACATTTCTCTTCAATTAT** | **(Chou *et al.* 2007)** |
| **VI (ATRW)** | **A/A** | **9** | **359** | **YM140463** | **ATRWTTAAATTTTTCACTTCTCCAACAACTATAAAGAGATCGACATTTCTCTTCAATTAT** | **This study** |
| **VI (ATRW)** | **A/A** | **9** | **923** | **YM140438** | **ATRWTTAAATTTTTCACTTCTCCAACAACTATAAAGAGATCGACATTTCTCTTCAATTAT** | **This study** |
| **VI (ATRW)** | **A/A** | **9** | **923** | **YM140458** | **ATRWTTAAATTTTTCACTTCTCCAACAACTATAAAGAGATCGACATTTCTCTTCAATTAT** | **This study** |
| **VI (ATRW)** | **A/A** | **9** | **923** | **YM140474** | **ATRWTTAAATTTTTCACTTCTCCAACAACTATAAAGAGATCGACATTTCTCTTCAATTAT** | **This study** |
| **VI (ATRW)** | **A/A** | **10** | **601** | **YM140019** | **ATRWTTAAATTTTTCACTTCTCCAACAACTATAAAGAGATCGACATTTCTCTTCAATTAT** | **This study** |
| **VII (ATWA)** | **A/A** | **3** | **370** | **YM140797** | **ATWATWAWWWWWTTYWYYWCTYCWMCAACWAYWAWRARRWSRWCRWYWYTYYTCWWYWAT** | **This study** |

Table S4 Logistic regression for type IV and clade 4

| **Variable** |  | **Clade 4 (n = 38)** |  | **Non-Clade 4 (n = 82)** |  | **P value** |
| --- | --- | --- | --- | --- | --- | --- |
| **Type IV** |  | **36 (94.74)** |  | **12 (14.63)** |  | **< 0.001** |
|  |  |  |  |  |  |  |
|  |  |  |  |  |  |  |
|  |  |  |  |  |  |  |
| **Variable** |  | **OR** |  | **95% CI of OR** |  |  |
| **Clade 4** |  | **105** |  | **22.28 – 494.9** |  |  |
|  |  |  |  |  |  |  |
|  | | | | | | |

Abbreviations: OR, Odds ratios; CI, confidence interval.

Table S5

Summary of the 161 *C. tropicalis* isolates used in this study from NCBI database.

| **CTRG_05978** | ***SNQ2*** | **MLST Clade** | **DST** | **SRR#** | **Continent** |  |
| --- | --- | --- | --- | --- | --- | --- |
| **Type IV** | **A2977G** |  |  |  |  |  |
| N | A/A | 2 | 203 | SRR18971070 | Europe |  |
| N | A/A | 2 | 134 | SRR23915273 | Asia |  |
| N | A/A | 2 | 1344 | SRR23915639 | Asia |  |
| N | A/A | 2 | 134 | SRR23915457 | Asia |  |
| N | A/A | 2 | 682 | SRR23915628 | Asia |  |
| N | A/A | 2 | 779 | SRR20760411 | Oceania |  |
| N | A/A | 2 | 238 | SRR12823735 | North America |  |
| N | A/A | 2 | 875 | SRR12823737 | North America |  |
| N | A/A | 2 | 237 | SRR20760337 | Oceania |  |
| N | A/A | 2 | 134 | SRR20760345 | Oceania |  |
| N | A/A | 2 | 134 | SRR20760388 | Oceania |  |
| N | A/A | 2 | 134 | SRR20760407 | Oceania |  |
| N | A/A | 2 | 134 | SRR20760392 | Oceania |  |
| N | A/A | 2 | 134 | SRR12823754 | Europe |  |
| N | A/A | 2 | 134 | SRR20760383 | Oceania |  |
| N | A/A | 2 | 155 | SRR20760351 | Oceania |  |
| N | A/A | 1 | 31 | SRR18971069 | Europe |  |
| N | A/A | 3 | 1211 | SRR12823714 | North America |  |
| N | A/A | 3 | 1232 | SRR10977169 | － |  |
| N | A/A | 3 | 1198 | SRR12823760 | Europe |  |
| N | A/A | 3 | 1198 | SRR12823757 | Europe |  |
| N | A/A | 3 | 110 | SRR23915651 | Asia |  |
| N | A/A | 3 | 1198 | SRR20760348 | Oceania |  |
| N | A/A | 3 | 1198 | SRR12823728 | North America |  |
| N | A/A | 3 | 1198 | SRR20760384 | Oceania |  |
| N | A/A | 3 | 1198 | SRR20760360 | Oceania |  |
| N | A/A | 3 | 862 | SRR20760349 | Oceania |  |
| N | A/A | 3 | 1198 | SRR20760359 | Oceania |  |
| N | A/A | 3 | 1198 | SRR20760374 | Oceania |  |
| N | A/A | 3 | 1198 | SRR20760367 | Oceania |  |
| N | A/A | 4 | 1000 | SRR23915286 | Asia |  |
| N | A/A | 4 | 385 | SRR23915267 | Asia |  |
| Y | A/A | 9 | 184 | SRR23915336 | Asia |  |
| Y | A/A | 9 | 184 | SRR23915559 | Asia |  |
| Y | A/A | 9 | 184 | SRR23915512 | Asia |  |
| Y | A/A | 9 | 184 | SRR23915626 | Asia |  |
| Y | A/A | 9 | 184 | SRR23915409 | Asia |  |
| Y | A/A | 9 | 139 | SRR18971071 | Europe |  |
| Y | A/A | 9 | 139 | SRR12823772 | Europe |  |
| Y | A/A | 9 | 139 | SRR23915501 | Asia |  |
| Y | A/A | 9 | 139 | SRR23915596 | Asia |  |
| Y | A/A | 9 | 401 | SRR23915485 | Asia |  |
| N | A/A | 11 | 750 | SRR12823744 | Europe |  |
| N | A/A | 11 | 359 | SRR12823712 | North America |  |
| N | A/A | 5 | 331 | SRR23915259 | Asia |  |
| N | A/A | 5 | 394 | SRR23915527 | Asia |  |
| N | A/A | 5 | 331 | SRR23915377 | Asia |  |
| N | A/A | 5 | 981 | SRR23915505 | Asia |  |
| N | A/A | 5 | 394 | SRR23915372 | Asia |  |
| N | A/A | 5 | 434 | SRR23915280 | Asia |  |
| N | A/A | 5 | 434 | SRR23915335 | Asia |  |
| N | A/A | 5 | 331 | SRR20760353 | Oceania |  |
| N | A/A | 5 | 443 | SRR23915306 | Asia |  |
| N | A/A | 5 | 1396 | SRR23915630 | Asia |  |
| N | A/A | 4 | 615 | SRR23915533 | Asia |  |
| N | A/A | 4 | 615 | SRR23915556 | Asia |  |
| N | A/A | 4 | 615 | SRR23915324 | Asia |  |
| N | A/A | 4 | 335 | SRR23915401 | Asia |  |
| N | A/A | 4 | 508 | SRR20760346 | Oceania |  |
| N | A/A | 4 | 508 | SRR23915491 | Asia |  |
| N | A/A | 4 | 508 | SRR23915357 | Asia |  |
| N | A/A | 4 | 508 | SRR23915358 | Asia |  |
| Y | A/G | 4 | 225 | SRR23915472 | Asia |  |
| Y | A/G | 4 | 225 | SRR23915353 | Asia |  |
| Y | A/G | 4 | 506 | SRR23915659 | Asia |  |
| Y | A/G | 4 | 506 | SRR23915675 | Asia |  |
| Y | A/G | 4 | 506 | SRR23915523 | Asia |  |
| Y | A/G | 4 | 225 | SRR20760377 | Oceania |  |
| Y | A/G | 4 | 506 | SRR23915528 | Asia |  |
| N | A/G | 4 | 506 | SRR23915547 | Asia |  |
| Y | A/G | 4 | 506 | SRR23915290 | Asia |  |
| Y | A/G | 4 | 506 | SRR23915522 | Asia |  |
| Y | A/G | 4 | 506 | SRR23915484 | Asia |  |
| Y | A/G | 4 | 506 | SRR23915385 | Asia |  |
| Y | A/G | 4 | 506 | SRR23915416 | Asia |  |
| Y | A/G | 4 | 506 | SRR23915276 | Asia |  |
| Y | A/G | 4 | 225 | SRR23915627 | Asia |  |
| Y | A/G | 4 | 506 | SRR23915482 | Asia |  |
| Y | A/G | 4 | 506 | SRR23915671 | Asia |  |
| Y | A/G | 4 | 225 | SRR23915261 | Asia |  |
| Y | A/G | 4 | 506 | SRR23915464 | Asia |  |
| Y | A/G | 4 | 506 | SRR23915283 | Asia |  |
| Y | A/G | 4 | 506 | SRR23915367 | Asia |  |
| Y | A/G | 4 | 506 | SRR23915425 | Asia |  |
| Y | A/G | 4 | 506 | SRR23915532 | Asia |  |
| Y | A/G | 4 | 225 | SRR23915631 | Asia |  |
| Y | A/G | 4 | 506 | SRR23915387 | Asia |  |
| Y | G/G | 4 | 506 | SRR23915302 | Asia |  |
| Y | A/G | 4 | 506 | SRR23915624 | Asia |  |
| Y | A/G | 4 | 225 | SRR23915487 | Asia |  |
| N | G/G | 4 | 225 | SRR23915668 | Asia |  |
| N | G/G | 4 | 225 | SRR23915670 | Asia |  |
| N | G/G | 4 | 225 | SRR23915667 | Asia |  |
| N | G/G | 4 | 225 | SRR23915299 | Asia |  |
| N | G/G | 4 | 225 | SRR23915663 | Asia |  |
| Y | A/G | 4 | 225 | SRR23915508 | Asia |  |
| Y | A/G | 4 | 506 | SRR23915494 | Asia |  |
| Y | A/G | 4 | 225 | SRR23915351 | Asia |  |
| Y | A/G | 4 | 506 | SRR23915518 | Asia |  |
| Y | A/G | 4 | 506 | SRR23915526 | Asia |  |
| Y | A/G | 4 | 225 | SRR23915594 | Asia |  |
| Y | A/G | 4 | 225 | SRR23915566 | Asia |  |
| Y | A/G | 4 | 225 | SRR23915568 | Asia |  |
| Y | A/G | 4 | 546 | SRR23915325 | Asia |  |
| Y | A/G | 4 | 506 | SRR23915365 | Asia |  |
| Y | A/G | 4 | 595 | SRR23915555 | Asia |  |
| N | G/G | 4 | 225 | SRR23915363 | Asia |  |
| N | G/G | 4 | 225 | SRR23915582 | Asia |  |
| N | G/G | 4 | 225 | SRR23915390 | Asia |  |
| Y | A/G | 4 | 225 | SRR20760369 | Asia |  |
| Y | A/G | 4 | 506 | SRR23915422 | Asia |  |
| Y | A/G | 4 | 225 | SRR23915392 | Asia |  |
| Y | A/G | 4 | 225 | SRR20760397 | Asia |  |
| Y | A/G | 4 | 506 | SRR20760358 | Asia |  |
| Y | A/G | 4 | 506 | SRR23915483 | Asia |  |
| Y | A/G | 4 | 225 | SRR23915550 | Asia |  |
| Y | A/G | 4 | 506 | SRR23915258 | Asia |  |
| Y | A/G | 4 | 754 | SRR23915294 | Asia |  |
| Y | A/G | 4 | 754 | SRR23915419 | Asia |  |
| Y | A/G | 4 | 506 | SRR23915310 | Asia |  |
| Y | A/G | 4 | 506 | SRR23915514 | Asia |  |
| Y | A/G | 4 | 506 | SRR23915232 | Asia |  |
| Y | A/G | 4 | 506 | SRR23915307 | Asia |  |
| Y | A/G | 4 | 506 | SRR23915378 | Asia |  |
| Y | A/G | 4 | 506 | SRR23915525 | Asia |  |
| Y | A/G | 4 | 506 | SRR23915661 | Asia |  |
| Y | A/G | 4 | 754 | SRR23915649 | Asia |  |
| Y | A/G | 4 | 506 | SRR23915458 | Asia |  |
| Y | A/G | 4 | 506 | SRR23915305 | Asia |  |
| Y | A/G | 4 | 600 | SRR23915362 | Asia |  |
| Y | A/G | 4 | 506 | SRR23915500 | Asia |  |
| Y | A/G | 10 | 343 | SRR23915634 | Asia |  |
| N | A/G | 7 | 983 | SRR23915266 | Asia |  |
| N | A/G | 8 | 439 | SRR20760366 | Oceania |  |
| N | A/G | 8 | 439 | SRR23915562 | Asia |  |
| N | A/A | 7 | 197 | SRR23915414 | Asia |  |
| N | A/A | 7 | 520 | SRR23915430 | Asia |  |
| N | A/A | 7 | 520 | SRR23915570 | Asia |  |
| N | A/A | 6 | 421 | SRR23915418 | Asia |  |
| N | A/A | 6 | 421 | SRR23915648 | Asia |  |
| N | A/A | 6 | 1361 | SRR23915360 | Asia |  |
| N | A/A | 6 | 1361 | SRR23915243 | Asia |  |
| N | A/A | 6 | 1361 | SRR23915349 | Asia |  |
| N | A/A | 6 | 1361 | SRR23915564 | Asia |  |
| N | A/A | 6 | 1361 | SRR23915375 | Asia |  |
| N | A/A | 6 | 1361 | SRR23915406 | Asia |  |
| N | A/A | 6 | 421 | SRR23915589 | Asia |  |
| N | A/A | 6 | 421 | SRR23915650 | Asia |  |
| N | A/A | 6 | 1361 | SRR23915579 | Asia |  |
| N | A/A | 6 | 421 | SRR23915509 | Asia |  |
| N | A/A | 6 | 421 | SRR23915369 | Asia |  |
| N | A/A | 6 | 421 | SRR23915370 | Asia |  |
| N | A/A | 6 | 421 | SRR23915368 | Asia |  |
| N | A/A | 6 | 421 | SRR23915552 | Asia |  |
| N | A/A | 5 | 678 | SRR20760376 | Oceania |  |
| N | A/A | 5 | 168 | SRR20760394 | Oceania |  |
| N | A/A | 5 | 678 | SRR20760390 | Oceania |  |
| N | A/A | 5 | 678 | SRR20760405 | Oceania |  |
| N | A/A | 5 | 678 | SRR20760365 | Oceania |  |
| N | A/G | 10 | 1378 | SRR23915340 | Asia |  |
| N | A/G | 10 | 848 | SRR20760417 | Asia |  |
| Abbreviations: MLST, multilocus sequence typing; DST, diploid sequence type; WT, wild-type; SRA, NCBI sequence read archive (https://www.ncbi.nlm.nih.gov/sra). | | | | | | |
